# Grounding and Applying an Ethical Test to Organisations as Moral Agents: The Case of Mondragon Corporation

**DOI:** 10.1007/s40926-022-00196-2

**Published:** 2022-08-15

**Authors:** David Ardagh

**Affiliations:** Charles Sturt University, School of Management, Wagga Wagga, New South Wales Australia

**Keywords:** Quasi-personal corporation, Corporate moral agency, Mondragon Corporation, Economic democracy

## Abstract

Moral people (i) have good goals in acting in a challenging situation; and (ii) use their rightly disposed intellectual and voluntary capacities (virtues) and resources to choose a good action in that situation. This requires (iii) sound ethical deliberation and decision-procedures for realising practically the abstract values and principles relevant in the concrete situation. After deliberation about sub-goals and means, they (iv) choose to execute the best particular action plan. They will have canvassed possible outcomes of the intended act, which, when executed, will have a particular result and (v) consequent outcomes for themselves, and for others; and (vi) it will show respect for the wellbeing of the community or wider society, represented by the state, and reflected in state law. A comprehensive third-party moral judgement on the act will encompass the intention and execution of the act in the situation.

Part 1, Sections I-IV of this paper argue that organisations can be said to be quasi-persons, personal and moral. As artificial organisational quasi-persons, with a similar structure to natural persons, they can also have analogues of the moral features of natural persons’ moral acts: i)-vi): good goals, rightly disposed capacities and resources, ethical decision procedures, good acts or activities, with good outcomes for others, and respect for society’s norms represented in state law. An ethical acronym, GREAOS, capturing the six dominant features shared with natural persons, is suggested.

In Part 2, an application of GREAOS is then made to assess a concrete organisational case, Mondragon Corporation, in order to show how if the analogy between persons and organisations is sound in principle, the acronym’s application can be made to ethically unpack a case. The acronym gives Mondragon Corporation at least a pass on ethics. Although a work in progress, its success in realising ideals of economic democracy can be an exemplar of stakeholder capitalism, an alternative to an unbridled shareholder capitalism, which has had its day.

**Supplementary Information:**

The online version contains supplementary material available at 10.1007/s40926-022-00196-2.

## Introduction

The connection of climate change to the global political economy of ecological degradation, business failure, unemployment and gross inequality of wealth, is being underlined by national lockdowns, in the name of the common good, to restrict the COVID19 virus spreading. In previous pandemics, like SARS and Ebola, and now in the time of COVID19, there has emerged the suggestion that those viruses which become pandemics, jump species, as a result of fires, or of environmental degradation due to deforestation. These are in turn caused by human induced climate change, leading to collapsing animal habitat.

Climate change arises in part from relentless human land-consumption, by mining and agribusiness; with fossil fuel usage creating carbons, or industrially farmed animals creating methane. These and associated ills, such as ocean-life depletion, through pollution, oil spills, or by long-line industrial fishing fleets, are associated with unlimited, profit-driven, global “shareholder capitalism”. Besides environmental ruin, the result of such shareholder capitalism over nearly three centuries has been excessive concentration of nearly half of human wealth in a tiny percentage of human hands, noted by Marxists (Wolff 2019), writers like Lundberg (1968) and more recently confirmed by Piketty (2017) and the Oxfam yearly reports (Oxfam (2019-21)). The 2016 Panama Papers and 2021 Pandora Papers disclosures about secretion of billions in tax havens have sharply exacerbated the global inequality issue. The Covid pandemic has brought the need for a public ethics of the common human good into focus. At the same time, there is new interest in “economic democracy”, including ethical reconstructions of shareholder capitalism as welfare or ‘stakeholder capitalism”. The waning of reductive individualism in ethics and economics converges with these two movements to favour revisiting collective moral responsibility in organisations.

These allegations have also sparked new interest in “wealth”, in the older literal sense of personal and social wellbeing, not mere capital accumulation. There is more interest in ethical curbs on the free market; and supporting improved forms of global now nearly universal “global welfare-state capitalism” (Nielsen (2003)). More radical forms of “economic democracy”, privileging finite human need and community-driven consumption and production, over boundless abstract corporate financial profit, suggest alternate forms of corporate governance to the older shareholder capitalist regimes, still embodied in Anglo-American corporate law. Many proposals, now featuring human need satisfaction, as opposed to mere de facto wants/preference satisfaction, and more need-based consumption and human control, are increasingly being discussed. (Cumbers (2020); Engler (2010) Harvey (2010); Barnes (2006) Clarke and Chanlat (2007); Smith (2005); Schweikart (2002) Dahl (1985)).

All these reforms attempt to give some sort of an internal “voice” to non-shareholders, and other internal and external stakeholders, like workers, suppliers, customers, competitors, communities, and representatives of the state. Input is allowed from special interest groups concerned with planetary environmental health, and, in general, groups not so focused totally on financial profit. Reformers challenge the view that “the business of business is business alone” within the law, as being amoral or immoral, and denying corporate social responsibility. This view is associated with some advocates of shareholder capitalism, like Milton Friedman, but it is opposed by many other theorists who recognise moral constraints on business and /or rights of other stakeholders. (Freeman, 1984; Freeman, Martin, and Parma (2019); Stiglitz (2019);Stiglitz, Fitoussi, and Durand (2019)).This is part of a 50 years of convergent Business Ethics literature on foundations of “corporate social responsibility”. It is also challenged by contemporary “communitarians” most of whom are following the doctrine of the common good, found originally in Aristotle’s *Politics*, and elaborated by Aquinas. Such challengers include Macintyre (Macintyre (1999)), Taylor (Taylor (1989)), Etzioni (Etzioni 2004; 2011) and Sandel (Sandel (2008)); together with some contemporary “social contract” theorists, such as Donaldson and Dunfee.(Donaldson and Dunfee (1999)) and social concession theorists like Dine (Dine 2000)).

The writers above reject “reductive individualism” in ontology, economics, and sociology. This is the view that collective entities do not really exist and act strictly speaking, but only their individual members: and consequently, as aggregates, they can have effects but cannot be moral agents. Margaret Thatcher’s statement in a 1987 *Woman’s Own* interview that there is no such thing as society, epitomises this position. Social groups are treated as aggregates, which emit causal changes in activity without being personal or responsible ethically.

To challenge this view that organisations cannot be moral agents, a number of Business Ethics academics were identified as holding related reductive individualist views by the author in three articles in the journal Philosophy of Management (Ardagh (2011); (2012); (2013); and developed in a further article in that journal (Ardagh (2017)) and a chapter in the Handbook of Philosophy of Management (2019a)). An acronym for ethically assessing organizations, GREAOS, based on a quasi-person model of organizations, QPM, was introduced in these articles. GREAOS is based on six similarities in the concept of personal moral action and organizational moral activity with respect to the goodness of their *goals in a situation*, G; *right exercise of capacities and resources*, R; good use of *ethical decision procedures*, E; good *chosen and executed actions*, A; with good *outcomes for others*, O; and conformity to *state-supported social norms and laws*, S.^1^

To promote an ethical shift to a common good and less individualistic approach, the first part of the paper presents an overview of the quasi-person model of organisations and of how using Neo-Aristotelian Philosophical Anthropology, Ethics, and Theory of Analogical Predication, a stakeholder conception of organisation can be shown to be preferable to a shareholder sovereignty conception, allowing the application of GREAOS. The Neo-Aristotelian account of human nature, general ethics of the person, and analogy of attribution have implication for special organisational ethics of organisations, as quasi-persons, and personal artefacts. Six common features of the moral activities of individual persons and their organisational analogues are identified. Both can exhibit good in their: (i) intended goal in a situation, (ii) exercise of rightly disposed virtuous capacities and resources, (iii) use of ethical decision-procedures, (iv) good execution of good actions, (v) concern for the outcomes for other parties, and (vi) respect for just social policy norms and laws of the state, representing society. An acronym, GREAOS, capturing the dominant element of each of these personal features by a letter, is constructed for ethical grading of moral activity.

In the second part of the paper the ethical acronym is applied to an organisational example: the Mondragon Corporation, (MC), in the Basque region of Spain. It is a promising candidate (a) to illustrate that an organisation, as a type of collective agent, can be ethical in many ways; (b) to link individual, organisational, and social ethics, not just in principle, but in six specific ways; and (c) to show that an organisation can be ethically assessed more comprehensively, by refence to the ethical acronym-GREAOS. It will be suggested that worker-managed, compound-board firms, like Mondragon Corporation, have the relevant six ethical features to a significant degree. To that degree, Mondragon could inspire a post-COVID ethical model for firms, facilitate economic democracy, social equality, and exemplify the “social concession” communitarians approach to business organisations. MC is chosen in part because it has explicitly aimed since 1956 to be ethical: to reduce economic inequality through benefit sharing; to increase worker information sharing; and give them control of decisions, via rules set down for admission, voice, and the sharing of benefits. It aims to promote the wellbeing of the community in which it operates. But it is lately accused of several ethical weaknesses which the acronym helps to pinpoint and record its attempted remedies for the problems.

To anticipate the outcome, like most other moral agents, MC will be found to be good, but imperfect. The acronym can help to explicate MC’s ongoing internal organisational ethics in some detail. It has varied with policy changes, especially in the last decade of overseas expansion, and in some ways, this has been for the worse morally. The GREAOS acronym is useful in sorting out what is good and less good in such an organisation, by its use to measure historical performance and the internal “parts” of a real collective moral agent, with personal incumbents in roles^2^. This replaces inferring a sweeping ethical judgement from observing tracts of external behaviour of the whole organisation, as an impersonal causal aggregate.

Part 1: I Overview of the Quasi-person model of organisation.

This model described in previous works listed above is based on a Neo-Aristotelian theory of human nature, ethics, and theory of analogical attribution. Organisations are not persons, and they are not independent substances, but they are beings and agents, made up of persons who are substances, and caused by persons to exist in some way. Organisational acts are caused by persons, so organisations are “personal”, and their acts can be good or bad. Aristotle explicates his theory of analogical predication with the different uses of the terms “being” and “good”. Both apply primarily to substances, but also in an analogous sense, to other “beings’ like actions, relations, qualities and quantities, times and places, all of which exist and can be good in a sense (*Nicomachean Ethics 10*96a,15-1096b3). Along the same lines, Aristotle also gives an example of the word “health” at *Metaphysics* Book IV 1003a16-1004a4. Health is a state of the body, and entities like medicine, diet, and exercise, are causes of health; complexion or urine, are signs, or symptoms of health; and strength or calmness can be an effect. They can all be said to be “healthy”, but not health itself or themselves in the state of health. Yet the attribution is not just metaphorical or poetic, since these elements’ presence make a real difference to the presence of health.

In like manner, organisations can be said to be “personal” qua signs and effects of persons, or to be artificial persons or quasi-persons, who exist and act, well or badly, but they are not persons or substances. The internal analogue groups relevant to the acronym letters G, R, E, and A, who act “organisationally” to choose and enact good goals, do so as analogues of individual persons with rightly disposed capacities, in organisational roles. They are role incumbents in a structure. They do this primarily through sharing, adopting, or enabling the specific goals, capacities, decisions, and directing acts of their internal leadership groups. These leadership role incumbents are mainly members of boards, issuing directives to operational managers and staff, enabled by suppliers or investors, within a unified structure. They act together for the good of members and end-users, have good outcomes for other organisations in the practice, and show respect for society at large, represented by the state. People in organised group structures operate “organisationally” within some part of the social space of regions, domains, sectors, institutions, and practices.

Organisational acts are distinct from, yet performed within, such social spaces. Organisations are distinct from social forms like families, or practices, but organisations can manage affairs for agents within those spaces which have a collective goal structure. Their acts take place in circumstances specific to the domain, sector, institution, or practice. In an organisation, the description of the “propositional content” of the leadership group’s goals need not vary essentially from that of a personal goal, just because the organisational goal is specific and joint. Most human needs can figure as both personal and organisational goals in some situation -knowledge, health, security, or work. Whose goal is involved, individual or group, need not change the nature of the goal at every level of specificity. Nor need it alter the sub-goals or means. It will do so at the most particular level, since the answer to the question: whose goal? in the organisational case, will be that of a group, and division of labour or separation of powers structure means that they will have different specific parts or roles in the organisational act, performed as different agents, executing means to the shared goal in that domain, sector, or practice.

The use of personal reason in understanding the goal, in directing other personal capacities and enacting virtuous means to it, in the organisational context, will involve a board and CEO, in role, as “quasi mind and will”, to direct managers and staff in role, to carry out organisational roles as analogues of human capacities. But this hardly invalidates the idea that role incumbents, like plain individual persons, know what they are doing, in finding or executing reasonable means to the goal in a situation, one which can be assessed. The primary personal paradigm case in the analogy is an individual person using rightly disposed capacities or virtues to achieve a goal and the organisational analogue is a group of rightly disposed role incumbents doing the same thing around a joint propositional content.

Methods of ethical decision-making and practice, the E in the acronym, will still be applying principles and precepts of general ethics of justice where available. Justice requires equal positive freedom to seek wellbeing and negatively escape harm and equal respect for persons and differences in desert and contribution. In the organisational special ethics or code of ethics/conduct, needed in a domain or sector, institution or practice, the special ethics privileges, and exemptions, based on a social remit or concession (Dine (2000)), will still be suspended rationally from general ethics. Regarding E, only the last step, of voting or consensus checking, and some rules of order about giving due notice of meeting, agendas, and speaking etiquette, need to be peculiar to the organisational context, not the concept of justice nor the method of deliberation about moral objects, ends and circumstances, also used in the personal ethics case. Regarding the letters A, O, and S, at step A, the organisational actions as executed, following authorised working operational procedures, will be joint actions, following the ethical decision made by leaders. They can be monitored by accountants and auditors as analogue of biofeedback. At step O, one can check on outcomes for others, which will include primarily organisational end-users of a service or product, as external stakeholders, and other organisations in the practice. At step S, one can check on conformity with and consequences for society as measured by compliance with the customs and laws of communities and the ideally just state. We now turn to a fuller description of the detailed steps in the argument for the above proposal.

II The “Natural” Person in Good Shape and the Ingredient Goods of Wellbeing.

In the application of analogy of attribution made above, the “natural” person, or non-organisationally situated person or self, is the paradigm case of an existent ethical actor, and the first term in an analogical relation, with the corporate “self” or quasi-person as the other term. Before transposing the elements of personal ethical action to organisations as artificial persons, which will be developed in the next section, one must assess the paradigm case of a natural individual human person’s ontological status and good. In the Neo -Aristotelian description of the individual natural person, this paradigm case of a natural human person is what Aristotle calls a substance-a “rational animal”. The good, for living rational animals, is wellbeing, a “pre-moralised” good state of any living being with characteristic human powers or capacities. It is assumed that the development or perfection of its ontological capacities is attainable.

In our pre-scientific, common-sense idea of humanity, our “folk anthropology”, our sense of what it is to be “a person”, we often presuppose that there is a describable human phenomenon, “humanity” manifested in each individual person. Aristotle’s Philosophical Anthropology tries to capture this human “nature”, which each person shares. He calls this set of defining capacities, humanity, a “second substance” because it is not an independent individual. Yet the instances of this secondary substance are prime individual substances. They can be said to be capable of attaining “its” (humanity’s) ideal good state of human wellbeing. Aristotle’s word for this state is *eudaimonia.* A word for this state of wellbeing or happiness is found in most languages. Some languages have a separate word for happy feelings of euphoria, and some include euphoria as an episodic part of the more on-going disposition of happiness or wellbeing. Aristotle lists some indefinite marks of wellbeing: its ingredient ends perfect the “higher” human powers; apprehend their highest objects; are more permanent; are wanted for their own sake; are found in activities capable of cumulative, pleasurable, development, and are associated with self-sufficiency.^3^

Many of human nature and wellbeing’s physical substructures, like DNA, neurophysiology, and many bodily autonomic systems, and most of its psychological properties and ingredients, have been described and explained by empirical science, over more than twenty centuries. We expect this to continue. But philosophers such as Husserl (Husserl (1971)) and analytic philosophers like Austin (Austin 1961) and Searle, (Searle (1983); (1992)) rightly insist we still need a pre-scientific account of the wellbeing of the human subject, a “phenomenology” of the explanandum to be explained by science. The point of the label “folk anthropology” is to suggest we need to acknowledge an enduring pre-scientific notion of humanity, human capacity, and wellbeing to be explained.

Philosophical Anthropology is not natural science, but it has largely followed Aristotle since his *De Anima* in the use of terms like “psyche”, “soul”, “human nature”, ‘potential/power”, and “capacity”. These concepts are tools we can still use to tidy up our common usage and our intuitions about what we experience, the human explanandum, before we try to scientifically explain human life, capacity, need, and activity. For Neo-Aristotelians, a human person is a unified system of capacities in relation, and has characteristic human abilities, with objects and ends, which have distinctive needs and need-satisfiers, or “goods”. Personal instances of these capacities vary with age, health, nurture, and history. To assess the morality of actions, we look for the individual person and type of act in the context, a human person or self, or soul-body composite, however further conceived. We also assess the abstract tendency of a type of action they have performed in a type of circumstance. We look then to assess how well this agent’s action-instance in these circumstances, uses her human capacities with respect the attainment of happiness conceived as a common human wellbeing made up of the specific goods of capacity, personal and collective.

Each capacity of the self has a defining object and an ideal end, or state of good operation. The capacity of eyesight for example has actual optimal vision of a colour field as its object and good/end, and light as a pre-condition; the mind has knowledge of truth as its object/end and true belief about the world based on evidence as a precondition. Likewise for the other senses, limb use, respiration and digestion, there are ideal objects, ends, and optimal states and conditions. The properties of the human capacities of the self are of broadly three types.

- Mental: the prime mental capacity is a human self-consciousness, or self, or soul, with a mind and will *directing* our ability to understand concepts and propositional contents, like “that the cat is on the mat”. Mind knowingly judges the truth of asserted propositional contents, like “It is the case that the cat is on the mat”. Will wants or commands the realisation or execution of the propositional content, as in “Would that the cat were on the mat” or “Make it the case that the cat is on the mat” if the content is seen as a good. If seen as harm/evil, it is to be avoided. Our selves, through choice, inform and shape an originally inchoate human wish or desire or will for wellbeing, conceived indefinitely, in abstraction from greater specification, by mind/intellect. (Austin, 1967). The above noted marks of wellbeing, like self-sufficiency or permanence, help in this specification task, focused broadly on human needs-satisfaction. Supportive mental capacities of imagination and memory can be used to specify this desire for wellbeing and define intentions. The object of mind or intellect is knowledge of truth, including about the true good. The object of rational wish or desire (will) is the apprehension and assimilation of the true human good, part of the universal good.^4^
- Sensory motor: a healthy human adult can consciously flex or direct active *operational* powers shared with other higher animals like those of organs of the five senses, exercise of flexible limbs, capacity to move, rest, or reproduce; and primarily through use of these capacities, we feel emotion and bodily pleasure and pain. Each voluntary capacity has a specific good, like good sight or taste or hearing; limb flexibility, and correlative sensation and sensory enjoyment. 5
- Autonomic-Bodily: a human has some *enabling*, largely nonvoluntary organic autonomic systems of respiration, digestion, elimination; and plant-like non-voluntary growth of hair, nails and skin. This set of subsystems also includes biological /physiological functions like balance, circulation, or immunity. Their substructures are progressively understood by medical sciences, but not Philosophical Anthropology.

For convenience I will type (a) capacities *directing* powers; type (b) *operational*; and type (c) *enabling*. The architectonic structure, with its decreasing voluntary control from a) to c), will be dubbed the “DOE structure”. The organisation will have an analogue of this DOE structure, as the next section will explain. Type (a) capacities direct; type (b) operate; type (c) enable (Ardagh 2014).

Our capacities have needs, and need-satisfiers, and rightly disposed perfecting capacities, or virtues, and they are interdependent, in a quasi-hierarchy, where type a) capacities can be said to control or “rule” others, in some specified ways, and be ruled in others, in different cooperative ways. A person is a quasi-cooperative with a) capacities which need virtues, like practical wisdom and self-control, to integrate and nurture the other capacities which enable them. No capacity is fully autonomous. As noted above, when the person or self’ knows/believes or wants, that some subject, S, has a property, P, the common propositional content, “that S is P” describes the propositional content of their mental state, whether a belief or goal. The prefix “It is the case that…”connotes a claim of knowledge/belief that S is P; and a prefix like “Would that ….”or Make it the case that... . connotes a goal-expression of a wish or command or will that S is, or is to be made P.^6^ Persons use these two interlocked master-capacities of mind and will, with the same propositional content, to engage, support, or be supported by other type a) capacities, like memory or imagination, and capacities of types b) and c). Type a) and b) capacities nurture and preserve the c) capacities at “lower” levels of composite human life, in some ways, and are supported by them in others. Martha Nussbaum and Amartya Sen helpfully set out the connections between needs-satisfaction and capacity development (Nussbaum and Sen (1996); Sen (1999)) although their list of capacities and needs is not ordered hierarchically and somewhat different from the above, in running a) and b) together in some places.

The three types of capacity of the flourishing person/self causally interact, ideally in a co-operative way. For example, a person uses type (a) conscious/mental/cognitive capacities, voluntarily *directs* and causally flexes the *operational* perceptual and mobility capacities of type b), as when we turn our head to look at something. Yet they are also passively receiving information, and/or satisfaction, from the latter flexed type (b) optic capacities, in a cooperative activity, and they also depend on other type (a) capacities of memory and imagination. Thus, we cognitively ascertain means to overall wellbeing, and avoidance of harm; and, through feedback from type (b) powers, protect and nourish the *enabling* type (c) capacities on which our life/existence depends. Co-operating levels a) and b), through assessment of biofeedback and response, protect and nourish type c) powers in the seeking of the master-ends of survival, perpetuation, and development. We will look for an analogue of this structured direction by virtue of need-satisfaction in organizations later.

In Aquinas’ version of Aristotle’s Philosophical Anthropology of capacity, type (a) capacities are ideally architectonic to (b) and (c) as agent causes. They ideally *direct*, nurture and deploy or dominate the focus of *operational* sensory-motor capacities, as efficient and final causes and nurture the *enabling* type c) autonomic bodily capacities. They keep the whole integrated human entity together to survive, self-perpetuate, and develop through social engagement with community/common goods, the focus of *Politics*. The type a) capacity of “will” is at first naturally focused on wellbeing, indefinitely conceived, but ideally gradually connects with the more specific “highest” goods-the true goods- those meeting the marks of wellbeing like delight. If some key type c) capacities fail for lack of nurture or otherwise, we die.

Good states of type a) capacities, are normally prior in finality, or “choice-worthiness”. In the order of voluntary execution, and of abstract “choice-worthiness”, we normally value understanding, friendship, and love (in capacities of type a), and sights and sounds, exercise, and play, (type b) over unconsciously assumed continuous physical existence or life at any cost. Non-resuscitation agreements attest to this. We can decide to amputate a diseased limb to save the whole person, thus privileging (a) and c) over some power of (b) and (c) in some respect. Care of type c) nurtures self-preservation of conscious life; self-perpetuation through children for the sake of type (a) and (b) capacities is also a main motive of humans.

Some capacities act on others in one respect, or are more choice-worthy, yet are passive or inferior to them in other respects. This architectonic inter-capacity relation exhibits a flat cooperative quasi-hierarchy. Type (a) and (b) capacities ideal object/ends as normally, but not always, more choice worthy. Only in some contexts and for some reasons, like breakdowns of (c) type functions, are their inclinations and directions to be outweighed. But which circumstances? For guidance we need rational personal ethics of self-care and care of others, in order to assess competing wants, needs, and goods of our own quasi-hierarchical person/body, for wellbeing, and a general ethics to balance this self-care with compassion for the wants, needs, and goods of others.

III The Moral Person: From the Anthropology of Good to General/Common Ethics and Justice.

This section offers a necessarily compressed account of the ethical decision-making method used in a Neo-Aristotelian teleological account of Ethics. It assumes right choice and right judgement are related to virtues of the human capacities, goods, and needs noted in the Philosophical Anthropology of Part 1.II above. The idea of a species-entity with a set of ingredient goods, which we all share in some indefinite way, as “wellbeing”, is presupposed by our moral assessments of right action. Ethics of virtue concerns which goods and whose good to prefer in particular real circumstances. The state of flourishing as a human, or being in a good state as a person, equated with happiness or wellbeing, is presupposed in our moral judgements of conscience regarding doing good for ourselves and others. We assess what is morally right in part by reference to rules about what act types tend, abstractly considered, to be worthwhile and promote this good state of wellbeing in our hierarchical capacities of common human nature, for ourselves and others, when they compete.

Moral persons wish this wellbeing to be acquired by all, themselves, and others. They realise the need to rank goods in the right order, privileging the object/ends and goods of “higher” type a) powers of the self, noted above, as *prima facie* preferable. They see that this requires personal moral virtues like practical wisdom, temperance, and social virtues like justice. One can choose to do something right and good for the common human good, in an interpersonal domain, sector, or practice, within one’s power, limited by their circumstances. This disposition or virtue is justice, expressed in general principles of interpersonal responsiveness, like the Golden Rule: “Do as you would be done by”. Some general principles, like: “Do as you would wish others in similar circumstances to act”, put the main emphasis, in the morality of justice, on willing universally, consistently, and impartially, and respecting the right to temporal happiness of others, without seeking more than what is due regarding one’s own wellbeing. Others see this moderate benevolence or altruism as also requiring one to promote the common good and long-term greatest wellbeing of the greatest number possible in the context, including the individual. Yet others, who are usually religious believers, also see the possibility of a transcendent wellbeing, extending beyond the temporal bounds of death, linked to a creative God, or more exacting rules of intrapersonal response like total nonviolence and forgiveness. This eternal happiness can often be linked to the notion that enlightened self-interest suggests that if we pursue or defend the wellbeing of others well beyond that of our own, or” beyond the call of duty”, eternal happiness can later follow in an afterlife or afterlives.

These three sorts of account of personal morality and justice, both natural and supernatural, moderate and strongly altruist, mostly overlap in their accounts of the core values, moral virtues, principles and precepts of communal/public morality. They diverge mainly in that the latter religious writers supply an avatar or prophet proclaiming some added transcendent dimension; and an added scope and motivation for going beyond the call of duty, aspiring for a more exacting, heroic moral perfection. Chisholm (1968) and Urmson (1958) distinguish moral modalities, like “supererogation” and heroic morality, from recommendations, duties, permissions, prohibitions and the execrable. 7 The accounts also overlap in the main on how to treat exceptions to moral rules and use of casuistry when rules collide. They affirm personal free choice and conscience. They differ mainly on the scope and motive of the above noted principles of inter-personal responsiveness, and on whether a response is in a moral modality like ideal, commendable, required or prohibited. We need not decide between them for purposes of establishing the existence of core or basic collective moral agency.

Principles, Precepts, Moral Notions and “Object, End, and Circumstance” Methods of Ethical Decision Making.

The most general moral principles or imperatives are abstract, such as:” Act justly”, or “Be reasonable” or “Avoid harming others”. Justice is about equality of respect for persons, equality of bounded freedom of choice, of opportunity, and of respect for their contributions. Aristotle treats exchange or commercial justice, distributive justice, and retributive justice as different ways of ‘giving everyone their due’, treating the same the same and the different as different. But he is discussing the best citizens of Athens, not slaves and aliens. He somewhat curiously avoids explicitly endorsing any other general interpersonal responsiveness principles such as: “Do as you would be done by”, or “Do as you would want others in the same sort of circumstances to act,” or “Promote the greatest happiness of the greatest number”, already mentioned above. The latter would all seem to be more morally compelling in 2021.

More specifically, there are specific, near-universal moral precepts, prohibitions against types of action in types of circumstances, picked out by what Kovesi (1967) called “moral notions,” like murder, theft, adultery, perjury, or cruelty. Such notions denote pre-collected clusters of “act-and-circumstance” type, of long-standing moral interest. Murder e.g. is deliberately killing an innocent person; adultery is sleeping with another person’s spouse. Where specific precepts and common moral notions are absent, or rules do not apply, we still have conscience, and methods of moral decision making, like casuistry, for addressing such complex circumstances.

Casuistry here is used as short for” the method of object, end and circumstance”^8^. It is a method of making decisions, using available precepts but not requiring a pre-existing precept or moral notion term like theft, or murder, or where there is a moral notion, but its application is complex. For example, in applying specific property rights in cross cultural situations to establish theft; or where precepts collide, or as with “life versus quality of life” issues, and the definitions of the concept of a person, in the abortion case. Applying special professional domain exemptions about not telling someone the truth, without lying, is a less clear case, but is similar. Casuistry helps to deal methodically with particular circumstances, to see if they fit the relevant moral notions. Casuistry suggests that the journalist’s questions: Who? (With Whom? To Whom?), What? Why? (For What? Where?, When/ and How? (How Much?)? are a good start to address, and morally assess, both the type of action and its type of circumstances. 9

In a Neo-Aristotelian Moral Philosophy, decisions using casuistry are made as follows.

1. In answering the Who? and With whom? questions, we look at identifying the relevant agent or agents. Answering the question What? identifies the abstract nuclear type of an individual action, the action’s object/end, expressed in a propositional content. For example, “that the cat is in the garden” expresses a possible end-result in view, in a propositional content, as described above. Prefixing: “It is the case ….to the that clause in this case gives us the assertion that the cat is in the garden. If true, we have the situation existing after any commanded successful action to bring it about as a goal is satisfied. If this were a goal to be adopted, we would now have the goal of the type of act, whose goal, and what goal. One could then assess abstractly its tendency to promote human wellbeing when executed. It will usually be neutral, as here, (the cat being in the garden). But sometimes it will fall under a moral notion or have a positive wellbeing related colouration and be prima facie good (that Mary cheers up); or a bad one (that a person be killed or a war begin ).
2. By adding the answers to the circumstantial questions: how? how much? when? and where? and other relevant features like to whom? or with whom? the now more specific act is ready to be assessed again. It now conveys the quality of the subordinate ends and means to the specific goal as contemplated. One can then ask of this more specific act-and-circumstance, the same question of tendency of the act in these added circumstances, to achieve wellbeing. This act-type, in the type circumstances, will allow one to say that it is *pro tanto* good, bad, or indifferent. There is now a higher probability of a *prima facie* answer on its moral status. But again there still may not be a definite answer, or there may not be a moral principle or precept to hand.
3. Next, the circumstances why? (end) and what? (object) are re-visited. The end result of the goal, as end/object in step 1. above, has already been canvassed (the cat’s being in the garden). We now consider the goal as the end in intention and motive of the agent (s) in doing the act in the added-circumstance, and executing the means to it, in all the circumstances. This now includes ulterior intent and motives, good and bad. We can infer from this whether it would be a moral act or exercise of a virtue.

Once circumstances at step 2/3- are supplied, if the act-circumstance type falls under a common moral notion like murder, theft, or lying, for which there is a prohibiting imperative precept, it is *prima facie* or *pro tanto* wrong, unless there are further extraordinary added outweighing considerations. Such rules, where present, are presumptively to be obeyed, but defeasible in some circumstances. One can possibly lie to an intending murderer about his intended victim’s whereabouts.

If, as more often happens, at step 2/3, there is no relevant rule requiring or prohibiting or commending the act/circumstance type in a given context, one looks at the tendency to wellbeing of that type of act/circumstance cluster, of abstract object/end/ circumstance, as descriptive of that particular case, now more specifically described. One can read off a presumptive moral assessment, without resorting to general principles. If at step 3.we look back at the intent (what) and end/motive (why) of this agent in performing this action here and now, in these circumstances- the what and why of this particular agent in doing this act in these circumstances, then that earlier presumption at step 2 might be overturned: a bad action might be excused; a good action might be seen as only a strategic means to a planned evil. But the judgement at step 3 is rationally based, and moral deliberation can be concluded.^10^

The preliminary answers to casuistry’s questions: What? Why? How? Where? and When? questions are directed upon, and relative to, the answers to the Who/with whom? questions. Once we have all these details, at step 2, we have a kind of *prima facie* or *pro tanto* characterisation of the goal and act-circumstance cluster, with its agent identified and its tendency and probable result. This is not yet a morally definitive characterisation, until we complete step 3.where we revisit what (object)and why (end) questions again, to look at this person’s actual intent in doing this act here and now in this manner- the ultimate description of the act in all the circumstances – for a judgement on its good and moral rightness.

In ethically assessing a person’s executed actions as good or bad, right or wrong, in General Ethics, one looks at what the person knew and wanted to do as an end or goal in the circumstances; at what they could have done by reasonably exercising their repertoire of virtuous capacity to find means to execute what the how, when and where questions indicated. What they ultimately intended and chose to do after ethical deliberation, having used an ethics decision procedure covering the steps above in some equivalent way is decisive. The ethical decision procedure can be continued or repeated to cover what occurs as a consequent outcome of their executed choice after deliberation in the circumstances. Here one examines the good or rightness of the action’s consequences, as disclosed by feedback; for other parties, and its conformity to norms of communities and society, as represented by state-law and social institutions. Even if the individual action is not in the public domain, it may be appropriate for the act to be assessed for its foreseeable, foreseen, and any unintended outcomes for other parties, and it may be permissible or prohibited by state law or institutional social norms. As noted above, the act may have many other moral modalities than right or wrong: heroic, excellent, praiseworthy, very good, neutral, alright, inadvisable, prohibited, and execrable.

In sum, a good person in acting morally has a good **goal in a set of circumstances**, including intended or planned sub-goals and means; uses **rightly disposed capacities** (virtues) in accord with right reason to makes a good ethical decision; knowing what they are doing and why. They know how to rightly exercise their repertoire of-virtuous capacity well, following use of good **ethical decision procedures** like the above to make good ethical choices. They possess virtues to help carry out the good, intended **action** well and respond to feedback from the results of the executed action. They consider its intended outcome before execution and probable **outcome for self and others**. They assess its conformity to norms of the wider community, and the community of communities in society, represented ideally by the **state-supported law and instituted social norms**. Their incentive to follow their moral judgement is that all members of the group or community, including them in the long run, will be better off if they act morally even though they may be able to be a “free rider” by cheating now. The bolded phrase are summed up in the acronym **GREAOS.**

The features GRE are logically connected to A as intended action. A as intended is just the concluding choice ending ethical decision procedure E, which has canvassed G and R. As executed, A is pivotal in possibly causing consequent features O and S. These possible consequences can be canvassed prior to A as executed, and may be foreseeable, but in my usage, they may not be directly intended. Only A’s immediate result, not its consequence, is directly intended before its execution, which may even partly fail, or lead to later consequences which may be unintended and unforeseen.

IV From General Ethics of natural persons to group ethics of artificial persons: Special Ethics of Organisations.

As explained in the Introduction, the quasi-person model (QPM) is correlated with what section II (p6) called the DOE structure corresponding to mental, sensory motor, and autonomic capacities of type a)-c). As personal capacities are directive, operational, or enabling, so artificial persons can mimic this structure analogically to cover the six moral criteria in the new collective context and make personal and moral attributions to organisations more intelligible and coherent. In the organisational analogue or quasi-person, analogues of type a) directive cognitive and voluntary capacities such as mind and will, memory, imagination, and conscience, are exercised by *directing* leadership groups, as quasi-minds and wills, like a board of directors and chief executive officer, with constitutive organisational master goals. Organised groups leaders have prime architectonic or hierarchical, authority in organisational acts, and so primary moral responsibility in any shared interdependent action. They design or inherit interconnected repertoire capacities in the form of role structures as analogues of type a)-c) capacities. Leadership group analogues of type a) capacity might also include conscience (Ethics Committee) memory/remembered history (Records) or imagination/imagined futures (Planning, or Research); conscious verification/review (“biofeedback”) provided by Accounting and Audit.

Sensory motor type b) analogue capacities of the organisation are exercised by compliant subordinate officers and staff choosing or executing the right subgoals and means and reporting up to the board or boards. The b)-type capacities-might be mimicked in financial control, production, human resources, or marketing in a role structure. Senior managers and staff *operationalise* the goals; and seek cooperative parties, as analogues of type b) individual capacities; and they locate analogues of c) capacities, like *enabling* investors and suppliers. Directive bodily causal control and cooperation in capacities of the person, in the organisational analogue, is matched with compliance by incumbents in management and routine staff roles with organisational leadership authority. Ideally the ethical organisation has a cooperative organisational “self” with self-control and conscience now replaced with leader group governance, authorised rules, and “bottom-up” compliance ideally, accepted by incumbents. These groups are ideally designed to mimic the limited causal interactive and moral control of the self’s DOE structure, with a joint goal, role structure, and ethical decision capacity which can be monitored by ethics committees, accountants, and supervisors. Managers and staff are secondary operational moral agents of the organisation, and suppliers and investors are enablers.

We would expect different degrees of moral responsibility to follow different degrees of authority as between the three levels in the DOE structure. As in the case of the particular person, the ideal architectonic control of higher directive capacities of type (a) over (b) and c), is limited in certain respects by mutual causal dependence, within the conscious mind, of mind, memory, and imagination, and between capacities of type b) and c), so in the analogue organisation, authority is ideally plural, and self-limiting or otherwise limited. In middle to large organisations, as Shann Turnbull (2004) suggests, separation of powers should be the norm, and compound boards.^11^ But some board will have “the last word” in an interactive procedure. General Ethics’ decision method of casuistry can simply be nuanced to take account of the group context and changed circumstances presented by organisational practice, such any practical norms of a domain or sector, in a Special (organisational) Ethics. The governing council setting direction is the primary responsible organisational agent.

In medium-to- large organisations, as opposed to small enterprises, the board usually *directs the operating managers and staff to engage enabling agents* like suppliers and investors, using the authorised procedures. Following deliberation utilising the authorising procedures and any other relevant legal or moral decision procedures, such as consulting the principles and precepts for assessing individual moral acts cited above, with some domain relative qualifications, the organisation’s members jointly act on the chosen decision of the directive leadership group via the relevant operational and enabling role incumbents. These acts of the organisation have immediate results for the organisation and its end users; and for other parties and longer-term outcomes for the community and the wider state. In compound board structures, one has the last word.

As personal moral excellence stems from conscientious application of General interpersonal ethics, so in organisations the leadership group must respect the same norms, plus those of the Special Ethics relevant to the organisation’s domain as specified by its goal. Unlike their legality, their moral authority comes from the morality of their joint goal and intent to serve the common good. It does not come just from their “self”-created, “self”-constituting or foundational identity or history, any more than in the case of individual natural persons. Applying the G in the acronym as first step, **ethical goals**, and sub-goals as means, in a situation. can ideally be co-adopted and enacted by joint authorised agents serving as rightly disposed capacities of the complex interactive organisational entity as analogue. General personal ethics grounds its Special Ethics.

At each organisational level, there are different degrees of capacities and moral responsibility. As analogue of the D in the directing, operating, and enabling DOE structure for natural persons, leadership groups, like Boards of Directors and CEOs, as quasi minds and wills, both set the prime goals and ideally are **rightly disposed role incumbents**, whose goals primarily direct the other agents which make up the complex organisational act serving its goal. Their own roles are artefacts of organisational design. They and their subordinates are persons in relations. Causal directive, and compliance operational, and enabling organisational relations obtain between groups of individual persons co-operating. These individual persons exist as prime substances and exhibit moral qualities. Organisations exist as secondary beings and exercise right moral dispositions of capacity rationally, primarily through leader groups designing, and all groups following, agreed authorised directives in role structures. Leaders, operatives and enabling groups; and third-party external observers, can apply GREAOS to organisational acts at the intentional planning and post executed stages. At the former intentional action stage, the consequent outcomes, picked up by the letter O, are only probable or foreseeable, not certainly foreseen. But plans can be ethical. After execution, their consequences can be assessed as being good or not.

To different degrees, members can follow **ethical decision-making procedures for ethical choices** and respond to the rulings in their conclusions made through their boards’ authorised procedures. Boards and CEOs can try to assure the organisation acts based on ethical choice, through ethical procedures, ethical codes of conduct and ethical culture. Their joint executed **acts** can be assessed as good or bad, moral or immoral, if one knows where to look for authorised leadership and role incumbents within its authorised role/decision structure, practice or domain, and enveloping communities. This enables the organisation, through accounting and audit, and outside observers, to mimic biofeedback and morally assess its action’s **outcomes for others**, like end-users, other players in the same practice; and its conformity with and respect for law and social and political impacts on communities and social groups, which can be monitored by society as represented by the **state.**

What organisations can do will normally extend some powers of individuals, by division of labour. Together, incumbents exhibit greater power and longevity in some ways than that of a person or role incumbent. Organisations as artefacts cannot eat or make love, but they can last for centuries, in a place or places, attain collective goals, like public health or science, or conclude peace or trade treaties, through their leadership groups. They achieve collective goals, and exercise right reasoning capacities in their organisational repertoire. It is a repertoire, not of well-disposed affections, but of “roles enacted rightly” by role incumbents, requiring their personal right dispositions as directing or compliant, directed, causes.

The GREA letters of the acronym pick up that all the internal stakeholders have some agency in the intentional act, up till the choice of A and its results, in the order of intention. Letter A covers the act’s intention and execution. After execution of the immediate results of A, agents have completed that act. But because the DOE power structure in the analogue does not extend beyond authorised intention and command to act, the Quasi-person model, QPM, requires a Revised Stakeholder Model, to apply at Steps O and S of GREAOS.

In the QPM model, an internal stakeholder is one having a claim on the organization based in justice. This will be based on being an active contributing and vulnerable party as agent of the architectonic tri-level DOE action structure. External stakeholders have a claim as a group entity “outside” the firm, caused a benefit or harm, intended, unintended, foreseeable, or not, to its freedom or equality, involving general commutative or exchange justice. It is not based on some direct structured action contributing to the organisation itself as a whole entity. Board leaders, officers/staff, or enablers deserve positive freedom and equality of treatment by the organisation in conformity to a variant of the general responsiveness principles motioned in section III (such as the golden rule of reciprocity), in virtue of (a) directing, (b) doing the work of, or (c) enabling the organisation, “inside” the firm. Outcomes for other parties like end-users, competitors,and fellow citizens are owed in justice as passive external parties or stakeholders.

**Fig. 1:**
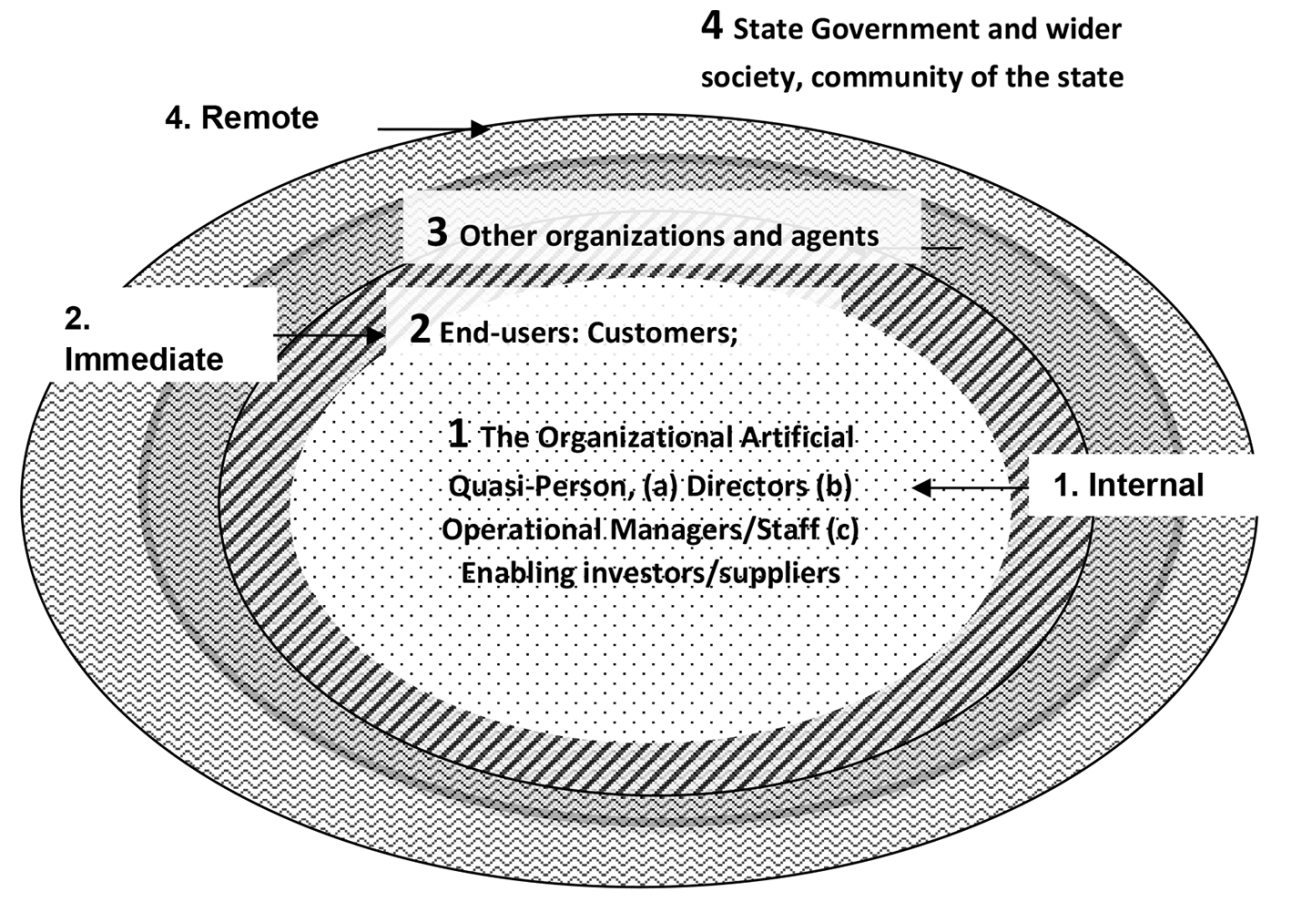
A Revised Stakeholder Model

In the Fig. 1, circle 1 shows three types of internal stakeholders who do the work cooperatively. Circle 2 represents end-users like customers or clients as in some way passive and external but intermediate stakeholders; Circle 3 represents other players in the domain or practice, those other than the inter-personally responsive conscious voluntary enablers, with contracts, like suppliers and investors. Circle 3 also includes such actors as independent researchers in the domain or sector, strategic developers, competitors, trade associations, peak bodies and industry self-regulators. Circle 4 includes the state, representing the wider society and communities as external stakeholders. They are remote from the actions of the artificial person but interact with it ethically.

After A is executed, we can ask, at GREAOS step O, as outside assessors, whether the consequences of A warrant that another organisational act should be performed in response. Accountants and other officials can focus on whether the verified outcome, the longer term consequences of organisational act A for the external stakeholders, were good and for whom; or whether there were any adverse impacts on other parties, beginning with the external stakeholders end-users, and then other players in the practice, and other external social and governmental stakeholders Under S we are asking about the impact on the wider society, community or the state was, and whether the relevant state or community laws were respected. Although self-care is part of morality, morality is also focussed on the way we treat other people when it is not in our immediate self-interest to do so. Both morality and law tend to reflect this factor. Organisations must monitor the same areas through their verification procedures- analogues of biofeedback.

The degree of moral responsibility is greatest for group 1(a). It lessens for managers and staff, investors, and suppliers. As one leaves the centre for the circumference, it lessens further. For small enterprises, some of the GREAOS features, like E, may be confined to a single person and so different e.g. for a single trader. But there will always be some analogue of the six features to be found in small organisations as well as states, cities, professions, business or education corporations, or non-profit secular or religious organisations. For example, taking G- goals and R-rational exercise of role-structure, in the public security, education, health, and welfare sectors, their goal is coping with ongoing and never-ending needs of citizens arising from human goals, specifying need-satisfiers for such needs of citizens, and the structure of organisational role incumbents is designed and entered at the top primarily by public credential or public vote on policy; or expert professional job qualified entrance at appropriate hierarchical DOE levels. As for persons, the organisational act can have different moral modalities from excellent to prohibited, not just right or wrong.

Part 2 Applying the GRAEOS acronym to Mondragon Corporation (MC).

I will now suggest that to a considerable extent the Spanish based Mondragon Corporation fulfils the promise of being an ethical organisation. In describing Mondragon as a changing entity over the more than 60 years it has existed, I will draw principally on the Mondragon-Otalora Cooperative Learning Centre website and the historical account of Barandiaran and Lezaun (2017) in “The Mondragon Experience” and their earlier “Humanity at Work: Corporate Management Model 2013”. 12 The history is also partly covered by other writers (Matthews, R (2012); Gollan and Patmore (2003); McMurtry and Reed (2009) Morrison (1991); Turnbull (2004); Whyte and Whyte (1991); Wolff (2016)).

In applying GREAOS, we will have a number of advances over other critics who pick one of the six features alone, in order to praise or condemn Mondragon, instead of a more comprehensive critique which locates the prime agency groups with a moral responsibility. GREAOS can show the relation of goals to structure over time and in different situations, and interactions with intermediate and external stakeholder, not just the observed outputs of an aggregated unit. Beginning in the late 1940s and into the late 1950s some employees of a local firm, some former students of local education institutions, and a priest trying to apply the papal encyclical *Rerum Novarum*, and some philosophical ideas drawn from the Neo-Thomist Maritain and Personalist Mounier, together with a savings bank, began a loose association of firms in the Arrasate region of Spain. After this, came the establishment of a consumer co-op, Eroski in 1969, an educational research co-op, Ikerlan, and one for professional services followed. The group expanded rapidly from the 1970s till the end of the last century. Since then it has grown into a large complex international entity, with revenue of up to 12 billion euros; 70–80,000 members, in 256 enterprises, of whom about 85% are members of the co-op, and about 15,000 workers (and growing) who are employees only.

### Goals

If we start with what goal and whose goal, the “Mondragon Corporation” is a changing entity with changing structures, but its identity is best found in its constitutive intentional goals which have guided and limited its structural changes, driven by core or essential ideals, not always realised in practice in all circumstances. I believe it is fair to treat it as one continuous organisational entity based on the egalitarian ideals expressed in its main documents and activities. These were explicitly formulated as ten principles around 1987 and have been preserved to varying degrees throughout its 60-year history, and even after its 1990s decisions to go international, changing its name from Mondragon Cooperative Consortium (MCC) to Mondragon Corporation, MC, and allowing foreign subsidiaries, which less strongly reflect or sometimes completely bypass its earlier cooperative structure.

As writers in support of the movement called “Economic Democracy”, mentioned at the start have observed, we are accustomed to the idea of political democracy but less accepting of that of economic democracy. Yet the goal of business need not be financial profit alone. As an organisation, MC adopts, as part of its constitutive goal, the language of participation in governance; member access to information, decision, and benefit- sharing. It sets up mutually limiting compound boards in its governance structure; and takes an explicit cooperative role structure as its form, with membership stakes replacing shares. It is a self-proclaimed economic democracy.

Mondragon is not unique in the sense that cooperatives are a very old idea (Tchami and Macdonald (2007)). Cooperatives function within states and state law, with a social concession or remit, a point also well made by Dine above. There are also large differences between Anglo-American and other cooperative governance systems (Clarke and Chanlat (2007)). The cooperative form is evolving and can be followed in globally read newsletters, such as *CoOp News*, U.K. and North America editions; websites like that of Cooperatives Europe, International Organisation of Industrial and Service Co-operatives, CICOPA; and the Co-operative Alliance. These websites offer global news for over a billion coop members and enable one to weigh up Mondragon’s distinctive features.

One important structural identity feature which was initially built into the goal and which we will revisit later under the acronym letter R is that the maximum size of any co-operative unit in the corporation was limited to 400–500 members. Thereafter, a new entity must be formed. In Mondragon, the goal of shareholder profit alone is replaced with entry-stake conditions of membership, and a percentage of profit sharing from the member’s own management participation and work. Mondragon Corporation, MC, is still basically a cooperative, with compound boards, directors and managers chosen by workers as members, and egalitarian values. It has been able to mimic analogically the balancing interactive DOE structure and quasi-communal, architectonically related, cooperative human capacities, in the “flat hierarchy” model, described in the case of personal ethical action. The size limit assures interpersonal contact. The secondary support coops like the educational arm providing knowledge and computer-based training; the bank and finance coops provide credit; and the outside support and internal unit social councils handle the material needs of members. There is still a directing leader group, the Governing Council, but it is chosen by members who do the work.

The description of the corporation relied on here applies less completely however to some of the co-ops which are parts of the post-1993 global entity, which, in going international to compete, has expanded enormously and in the process has dropped the requirement that all but 10% of staff be members of the cooperative. It now allows simple wage earners into operations outside the Basque region. Mondragon’s detractors call this “Co-op Capitalism”, and it forms the basis for the critique of Mondragon as a myth or an entity which has lost its moral compass. The scepticism is understandable, and I will address it again below, but what I will initially describe is the basic ideals of the entity, which has existed from the early 1960s, and reached a stable domestic stage in the years up to about 2004-07.

First, the post 1960, and more clearly the 1987–2007 entity, has some explicit and still extant “values” and “principles”. The stated corporate values of MCC were stated as: cooperation; participation; social responsibility; and innovation. The principles were:

1. Open admission to any who accept the values and principles.
2. Democratic organisation, allowing a voice to all members.
3. Sovereignty of labour.
4. Subordinate nature of capital. This is the correlate of 3. Above.
5. Participatory management where managers are assessed by workers.
6. Payment solidarity, or profit-pooling.
7. Inter-cooperation of co-ops.
8. Social transformation.
9. Universality in spreading social justice beyond borders.
10. Education for worker and youth.

These are the values and principles which are published and registered with a Council of Cooperative Groups, and when we apply GREAOS they supply an account of the intended master goals of Mondragon, our G in the acronym, along with the more specific constitutive sectoral goal of each member cooperative. Each unit cooperative has its own Social Council focused on member needs, management of their pensions, and other welfare matters.

Mondragon’s main business sectors of operation or sub-goals to meet the above master goals since about 1987 are: Finance, Industry, Retail, and Knowledge. Corresponding to finance as a sub -goal Mondragon has its own bank, Caja Laboral, insurance, and building and business consulting; Industry covers its wide range of coops producing items with innovative design, and the making of capital goods like robotic tools, auto-parts, and consumer appliances: Retail includes their extensive chain of Eroski supermarkets and selling of innovative products; and Knowledge covers a suite of Mondragon University activities, including training, research and development ( Ikerlan), and degree educations. If we try to evaluate the morality of its achieved goals and its intended goals, Mondragon *prima facie* would so far receive a good moral grade.

**Rightly disposed capacities**: To approach the more specific sub-goal profile we have to meld with the next letter, R, for analogues of rightly disposed capacity (virtue), like repertoire, or role-structure. In the organisational design of the unit cooperatives, membership is not by purchase of shares but entry fees and direct payment to a capital account, within the cooperative, as specified by the Governing Council. Distribution of profit is as followa:10% of profits are saved for central coordination of all co-ops;40% goes back into the enterprise;45% is distributed to members as a credit; 5% is dispensed in annual bonuses as additional salary, and there is a complex points system to assist in managing probation, training and transfers of any redundant member-workers to other locations, if they desire to move and to support them if not. Jobs are not just a cost to be reduced as in Anglo-American corporate practice, but the point of MC is to provide and retain good jobs. On leaving the coop or retiring they receive an amount back, social councils are a branch of the organisation dedicated to improving their financial position for when this time comes.

The basic structure is one where multiple departments, or units, organised geographically or divisionally or functionally, report to their Management Council, similar to a board, like an executive leadership team. They are under a Managing Director, like a CEO, selected by the Governing Council, acting like a supervisory Board of Directors, and all are ultimately subject to the General Assembly’s annual meeting and approval. The Governing Councils are elected by that General Assembly, and every member has a vote, all workers can play a part however small in shaping the direction of the producing whole. Social Councils are elected, operate like unions of worker-owners with advisory access to the Governing Council, and can bring an issue to the General Assembly. There are Audit Councils to monitor accounts. Some co-ops, like banks and health councils and research and development cooperatives serve and/or monitor others. There is inter-cooperative cooperation on many levels.

The compound board structure incorporates the values of cooperation, and participation, and the principles of worker sovereignty; the subordination of capital to labour; and democratic participatory management. These ideals are embodied to some degree in the role structure in that all members have some voice. The Social councils have rights of access to the Governing Council. This is an analogue of the cooperative mutually limiting personal capacities we saw in an individual, with the Governing Council, as ultimate cognitive and volitional authority, controlling all things in some ways; but subordinate to other entities in other respects, for example being controlled in others by the Social and Management Councils, and the general Assembly. Mondragon as a whole services many of its own needs from its own Bank, University, and support coops.

Higher level supervisory bodies are also subject to General Assembly approval, approving the goal-direction and sub-directions of the whole Mondragon entity, somewhat reminiscent of the personal mutual dependency of personal capacities we mentioned above. There is a set of overarching supra-cooperative governing bodies in the Mondragon Group of individual coops, guided by the Mondragon Corporate Model. They include the Cooperative Congress, which meets every four years. There is also a Standing Committee, elected by the four divisional Councils (Financial, Industrial, Retail, and Knowledge) which implements its resolutions. There is finally a Cooperative General Council which acts as executive of the whole Mondragon Group, and though it has no counterbalancing Social Council, it is charged with furthering internal solidarity between workers, councils, divisions, coops, and functions. So the compound board structure at unit and group level mimics the natural person’s complex interactive mental a) type capacities and DOE structure with top and intermediate elected leadership groups as capacities of the corporate body’s “self”- mind/will having limited rule authority over other mutual dependent capacities/role groups, which we saw was beneficial for personal moral agents.

The “self”-presentation of the 2008–2020 entity on the world wide web by Mikel Lezamiz and other representatives, still insists its original values and principles are implemented for members, while conceding they do not cover non-members. It lists the values and principles as ideal, and make at least symbolic moves in this ideal direction, even though they do not or cannot enact them in many of the MC overseas operations, whether US capitalist or Chinese socialist, and MC does not proselytise its ideals in overseas sites. Beyond the US and China, overseas countries where production and/or sales centres exist now also include Mexico, Portugal, France, Poland, Brazil, Germany, Czech Republic, UK, Italy, Romania, Turkey, Slovakia, India, Thailand, and Morocco. Although the account is morally impressive, this change in structure suggests we suspend moral judgement on the new entity for now. We turn now to acronym letter E.

**Ethical decision making procedures**. Beyond the values and principles above these cannot be known in detail, but must be inferred from the historical record, which has not been marred by major scandals like those of Enron, and positively has achieved great benefits for all its workers who are members and the communities in which it operates. Fr. Arizmendiarrieta, the founder of Mondragon, and part of the directorate for about 20years, would have been acquainted through seminary with Thomistic moral philosophy, and Maritain. Thomism closely follows the rationale and steps outlined above, on the moral person, including the casuistic method of determination of a conscience judgement. He at least would have set an example of such an ethical procedure and could well have assured some such ethical rationale and group casuistry decision procedure was being used.

The basic organisational decision procedure is broadly as follows. The Governing Council is elected by the members of the General Assembly, consisting of all members, with one vote per person, for a four-year period of office. A fixed ratio, on average about 1: 6, in size of pay, limits inequality between top directors and lowest paid workers, as opposed to 1:300 or more in the Anglo-American sphere. As noted the maximum size of each unit is 400–500 and when reached another unit must be started if needed. This is one of several conditions which are waived in overseas ventures in nations listed above, along with the membership requirement. About 85% of management and staff are members, and the other 15% and rising, are employees. Turnover is in the billions of dollars, and profit was about 160 million euros in 2018, as published in an Annual Report. Some other later estimates of revenue are in the billions of euros. The impact of the corona virus is bound to be enormous but is not fully known at this time (11/2021).

Applying the G, R, and E in GREAOS, one can locate constitutive and operational goals, roles, and ethical decision procedures in every operational and enabling unit by reference to the Management Councils’ different statements of specific mission and goals. In the non-international entities this will more strongly reflect the old MCC values and principles. None of MC’s joint goals are ethically suspect, and none entail a breach of any moral rule or precept. As to role structure within and between units, there is some effort to design a cooperative structure even in international units, though it falls well short of full worker management and ownership. The overseas workers who are not members still receive the normal benefits of employment.

**Executed Actions**: we turn now to A, action. As noted above, in personal morality one attempts to take account of one’s needs and those of others by following principles and precepts and value based moral imperatives about justice and personal virtue; and assuring that maximal knowledge of truth about the goods of wellbeing informs one’s desires and coordinated and cooperative actions, to produce overall benefits of wellbeing for oneself and others. The plan of action of A appears twice: first as an end in intention in the prior ethical decision making procedure or process, and later as a resultant executed act, to be then the subject of evaluation of its outcome for others, under O in the acronym. In the actions of the MCC organisational analogue, 1956–2021, there is explicit invocation of justice and shared information, shared collective ethical decision-making, for the purpose of shared benefit by members, although less for non-member employees.^13^ Organisational design and ethical procedures have been used to operate effective structures relative to their stated values; choice of good goals, and their resolve to deliberate on and choose ethical action.

### Outcomes for others

the values, and principles numbered 6–10 above (p 14) under G are all about the impact on others. Mondragon measures up well regarding successfully executed moral actions and the achievement of benign outcomes impacting on others, the O test. There is strong application of service to end-users, the welfare of the community and others in the practice. As far as the historical record at least up to 2019 goes, Mondragon measures up quite well. For example, in its support for workers made jobless following the bankruptcy of one of its largest cooperatives, Fagor Electodomesticos in the 2008 recession, the Audit or Watchdog Council assured there was a high degree of respect for ethical goods-like retrenched worker welfare through Lagun Aro, its health care and pension arm. Support for community development in University education and innovation, and the successful provision of products and services for end users over 60 years through the operations listed at G above, are relevant here.

### Social norm and State law compliance

As noted, social investment in the community is an explicit objective, and social welfare of members in domestic communities in which MC operates, has been well secured. There have been no reported major illegal practices or deviances from state laws. Of note is the endorsement of important global economists like Joseph Stiglitz reported in *Cooperative News* of the ratio of salary between CEOs and workers, a point also made by Wolff (2016) and McMurtry and Reed (2009). This has an exemplary effect on other corporations to hold down inequality and citizen alienation from the State. Its respect for national law is confirmable, despite the conflict between the Basque separatist movement ETA and Madrid and the terrorist incident in Madrid station in 2004, attributed by some to ETA but by most to al-Qaeda. So at first sight, Mondragon gets a pass up till 2019 on the S criterion, good political outcomes for, and conformity with laws of the state. So far as intended and executed moral action on the six features Mondragon seems to get a good grade. But it does have some serious omissions to account for in the ecological sphere in that its consumption of resources and carbon footprint are in no way exemplary. And that brings us to the critics.

Mondragon’s Weaknesses and Partial Moral Failures 14

With the explosion of international involvement from the 1990s onwards, many academics have been divided over whether and to what extent this favourable assessment can still be justified. If we use GREAOS to collect them here.

Re G: Even in the intended goals, there is a notable silence in the values and principles listed above on the environment and climate change, which we have now to acknowledge. None of these feature any explicit ecological goals. In this historical blindness, Mondragon is not by any measure alone and not the worst offender if one thinks of the multiply offending ecologically devastating Big Miners, especially of asbestos; or the Exxon Valdez and BP Gulf of Mexico oil-spills, or the devastation caused by agribusiness deforestation, nuclear tests and other armament makers, or Union Carbide in the Bophal case. But the point is still well made. Some of the industrial enterprises even in the original model are resource-intensive and ecologically dangerous in the climate change context.

Re R: This is the weakest link in the moral chain of MC. We suspended judgement earlier on MC as expanded into the international arena, around 1993.Its score even on execution of some of its own intended ethical goals, values, and principles, does indeed decline. Some of its international ventures, not requiring membership from workers, in the bid to be globally competitive, seem to be only slightly distinct from those of other large capitalist multinational business corporations, (Chomski 2014).In the present entity’s overseas operations, and a few local ones, now employing thousands of non-member workers, these are just wage earners. In the case of Poland around 2011, with local low wages, workers were not as well paid as other workers in Mondragon’s capitalist competitors. These workers are not benefitting in distinctive ways from membership of Mondragon.

RE E: We do not know how closely leading managers are following ethical decision procedures, although there are no scandals of any magnitude. The model, even without the expansion, has inherent dangers of de facto collective egoism, and manipulating of admission to increase existing member power and further their interests. The Basque culture may be necessary to lessen this temptation. If there have been moral execution failures, in the expansion, we can infer the failure of ethical decision methods.

Re A: In executed action, the co-op is allegedly not pro-active in gender equity matters, especially in retrenching casuals in order to cope with global downturns, and in practice Mondragon is still dependent on capitalism for import of innovation.^15^All non-member workers are excluded from the benefits of members and their number is approaching 20% of the total or higher, in some cases. Many unit cooperatives are ecologically hard on the earth’s health, e.g. those in the transport sector.

Re O: One could argue that the facts in G, R, E,and A above about the post 1993 expansion entail failure to treat non-member “others” justly, or equally. There would also be failure at step O.

Re S: The negative ecological points in G above put downward pressure on communities and States trying to cope with the climate crisis. The globalisation of Mondragon shows the inescapable tie to the global shareholder capitalist order and market driven global political economy.it may detract from the amount of benefit to the wider society, especially the Basque region, and the Spanish state’s financial benefit, if taxes are paid outside Spain. The whole socialist movement towards radical social reform of politics is blunted by this attempted two-tier hybrid.

Brief Responses: Some would say the criticisms show that Mondragon ideal is dead and gone, others that we have a two-tier entity, and some hold MC is an imperfect work in progress, but that the ideals are still in process of realisation. We saw above that there are multiple moral modalities of good and evil. The best and the good are different. Amongst the proffered defences of Mondragon, which are not presented as adequate, but suggest that MC is responding to criticisms, are the following:

G: Mondragon Corporation was originally, and, quite innocently, not committed to global social transformation, but only to that of the Basque region at best. Yet it supported the Kyoto protocol of 1997.Since global ecological consciousness has improved, from around 2012–2013, there have been some specific environmentally targeted projects in waste management, water management, and energy transmission, mentioned in the MC website’s own home presentation, even if environment is not featured in the original constitutive principles. There has been a move to address climate change issues and practical technologically based steps listed on the main website.

R: According to its own website sources, some serious efforts were made to introduce the basic model into its subsidiaries, at least as an option in so called “mixed cooperatives” and the promotion of what is dubbed “the corporate management model”, as an ideal. Both local and overseas workers themselves in some cooperatives have little zeal for or have explicitly rejected offers to take up full memberships of MC. After the collapse of Fagor Electroosmosis’s, with thousands of job losses, in the GFC economic crisis of a decade ago, the choice was seen by MC leadership as “expansion or non-existence”, and internationalisation allowed the survival of the Mondragon Corporate Model in some form in some places. Mondragon survives. Offers of full membership have been made difficult by the overseas countries’ legal arrangements or refused by workers unions in that country. Mixed models, deploying adaptations, such as reduced mandatory involvement, risk, and partial voice, have successfully adapted to the difficulties (Ayo and Alonzo, (2006)).

E: There is little evidence of internal manipulative exclusion from membership by incumbents in the suggested way. The success of MC mixed models outside Spain suggests Basque culture is not necessary for its ideal structure to work or be exported, not to work to be exported in great part. Some benign influencing on the capitalist economies, in which implementation of the model is not immediately fully possible, is actually happening and worthwhile, even if this is an imperfect outcome. Even some Marxists welcomed it as a progressive model. Its success in efforts to be socially responsible and just should not be forgotten because of a present partial failure of social transformation.

A: Governance and technical innovations are precisely what Mondragon embodies, and “technical innovation” is now explicitly one on the four values listed above as a dedicated objective of Ikerlan, the research/education arm, in the Mondragon website self-presentation. It is funded by their bank and promoted in their business consultancies. In many spheres of action, it has produced innovations of its own. The proportion of women members and employees has risen to around 43% according to Wolff (2016), and there are cooperatives within the Mondragon Group advertised on their website working on alternat energy, renewables, and waste management.

O: The points made about the past gender inequities and non-member workers mistreatment have been addressed to some extent and the ecological consciousness and gender equity profile of Mondragon has improved (Wolff (2016) (2019)). It still beats BIG Oil, Big Tobacco, or Big Mining in outcomes for others.

S: There has been some recognition of the need to fund the spreading of the model overseas though education to overcome overseas resistance from shareholder capitalist advocates and firms and Marxist, union, and “class-conscious” socialists. Since Covid, it has responded vigorously to the pandemic though its university educational and training capacity. There is no evidence of detriment to the Basque region or Spain.

## Conclusions

This cursory application of GREAOS shows that from a moral point of view, MC, like any organisation, can be morally assessed. As it is more difficult to describe a person as immoral than their specific action or actions, giving it an overall moral grade is challenging, but possible, and largely positive. It behaves as a moral agent capable of assessment, as ideal, good, neutral, or delinquent on each GREAOS feature and has good and bad performances, like that of a natural person. Without quantifying the assessment, its environmental footprint is ordinary, but it is improving. Until we more radically reform the now nearly global “welfare state capitalist” model, perhaps at G20 or UN level, our least bad option to avoid planetary despoliation is to move in the direction of cooperative forms of economic democracy. This means giving both internal and external social and state stakeholders, including natural scientists, some systematic consideration and voice and respect as a stakeholder, either in corporate decision making and sharing of benefit, though elected compound boards and general assemblies, or accepting social input from states, and other vehicles of economic democracy.

As measured by GREAOS, the six negative points, under the acronym, are still significant. MC is in some danger of losing its moral way in its overseas operations, where membership is not required. Yet it shows responsiveness to critiques, and sees itself as morally indebted to workers, end-users, other practitioners, and local communities. It acknowledges the law and its social concession to operate, in many ways like that of a profession. It takes its moral and social duties to change past failures seriously. The GREAOS acronym test reflects the quasi-person model of organisations and picks out organisational leader groups as prime organisational moral agents with goals, to whom intentionality and claims of justice can be attributed to prime degree. It also locates other groups like workers as responsible to lesser degrees for justice as presumptive equality of information, decisional input, and benefit from an enterprise. It reflects the dependence of special organisational ethics on general interpersonal ethics; and the warrant for our familiar descriptions and discussions of businesses and other social group organisations, as being wise or just, wrong or irresponsible. Such talk is coherent and reasonable, in acknowledging external stakeholders like communities and states as having some ethical claim, including the making of claims on behalf of the planet with which this paper began.

## Electronic Supplementary Material

Below is the link to the electronic supplementary material.

Supplementary Material 1

## Footnotes

1 In previous versions of GREAOS the letter R was sometimes also used to denote “repertoire” and “role”. The underlying idea is virtue, but “organisational virtue”, although analogically sound, was formerly thought to be prima facie too confronting and confusing. The letter O could also stand for other parties, and S could also stand for society, not the state,

2 The paper does not discuss many other, efforts to reform capitalism, such as that of Robert Owen in the 19^th^ century, Cadbury in early 20^th^, and Semco, Brazil or employee stock option plans today.

3 Aquinas collects and documents all these marks from Aristotle’s works at Summa Theologica, Part 1-IIae 1–5 and Part II -II Q.182,1. Aquinas lists the identifying marks of wellbeing as including self-sufficiency, ultimate finality, delight, permanence of access, leisure, and immanence. See also his version of Aristotelian Philosophical anthropology at Summa Theologica Part 1, Questions 75–89.

4 The universal good is the good everywhere. There is also a highest good.

5 Some scientists have posited a separate power of “intraception”, or “interoception”, monitoring emotions, but it is treated here as a species of the mind’s self-conscious attention.

6 This analysis depends on Searle’s Speech Act Theory (Searle 1983) and use of “folk psychology” on Searle (Searle (1992).

7 For further discussion of this meta-ethical account see Ardagh D.(2019b) Chaps. 1-5.

8 This account recognises that casuistry is also used in another sense to mean deliberately deceitful or evasive reasoning.

9 Summarised as “the method of object, end, and circumstance” by Aquinas 1a-11ae,7, and qq17-23.

10 This account of casuistry is set out in Summa Theologica Ia-11ae 7,and qq 17–23 and further discussed in Ardagh (2019b) Chap. 5.

11 See on this Shann Turnbull in Turnbull (2004). Architectonic relations are discussed in Ardagh 2014

12 available through www.mondragon-corporation.com.

13 on these last three goals, sharing information, decision making and benefits, see Kong, E, Jenkins,S., Ardagh, D. (2009) Vol 16, #6: “A Preliminary Analysis of Learning Processes for Innovation in Member-Serving Non-profit Organisations: the Role of Governance Structure and its elements–Shared Knowledge, Decision-Making and Benefit Sharing “*International Journal of Learning*, pp1-16.

14 for critical, mixed, and only partially favourable commentaries see Kasmir (1996) Bamberg (2017); Cheney, Udaondo, Etxagibel (2020) Ellerman,(2015); Flecha and Ngai (2014).

15 For responses to critiques see a reply by Ignacio Santa Cruz Ayo and Eva Alonzo, “Mondragon’s Third way: a reply to Sharryn Kasmir” in *Global Dialogue* magazine Vol 10, #3.(2016).
