# Supplementary material for: Grounding and Applying an Ethical Test to Organisations as Moral Agents: The Case of Mondragon Corporation
Source: Philos Manag. 2022 Aug 15;21(4):465–91. doi: 10.1007/s40926-022-00196-2 (PMC9377292; doi:10.1007/s40926-022-00196-2)
Supplement: Supplementary file 1 — Supplementary Material 1 [file 40926_2022_196_MOESM1_ESM.docx]

Reviewers comments response

I found both reviews to be careful, thoughtful, helpful, and constructive.

Reviewer !

I have attempted to explain what is distinctive about the acronym as requested and avoided the suggestion that the paper is extrapolating from Mondragon a theory about the wider role of cooperatives

I have tried to clear up the numerous typos and adopted most of the recommended changes on the original concerning font, capitalisation, conformity to guidelines etc; cut out the provocative asides, and removed the inconsistency about workers and membership

I have rewritten the section on the organisational analogy.

Reviewer 2

I have tried to improve the flow with shorter sentences. In the 5 previous articles I dealt with many aspects of the literature on corporate social responsibility, and have acknowledged its existence more clearly, and tried to make the Aristotelian theory of analogy of attribution a bit clearer and more focal earlier in the paper.I have minimised outside references to other philosophers who might distract from the Neo-Aristotelian approach taken, and tried to standardise the reference to each letter of the acronym. I include many footnotes in the text as suggested.I tried to clarify the connections of a)-c) capacities to the DOE structure. I also cut down on unexplained acronyms

P2 The two Abstracts resulted from my inability to delete the first one. I thought I has deleted it but evidently not

P3 The xxxxxx ing was supposed to respond to the anonymity requirement.

I am unsure if I have corrected all the spacing errors in the references list but I have tried.
